# Selection of Exopolysaccharide-Producing *Lactobacillus Plantarum* (*Lactiplantibacillus Plantarum*) Isolated from Algerian Fermented Foods for the Manufacture of Skim-Milk Fermented Products

**DOI:** 10.3390/microorganisms8081101

**Published:** 2020-07-23

**Authors:** Nadia Bachtarzi, Immacolata Speciale, Karima Kharroub, Cristina De Castro, Lorena Ruiz, Patricia Ruas-Madiedo

**Affiliations:** 1 Department of Microbiology and Biochemistry of Dairy Products, Instituto de Productos Lácteos de Asturias-Consejo Superior de Investigaciones Científicas (IPLA-CSIC), 33300 Villaviciosa, Asturias, Spain; bachtarzi.nadia@gmail.com (N.B.); lorena.ruiz@ipla.csic.es (L.R.); 2 Laboratoire de Recherche Biotechnologie et Qualité des Aliments (BIOQUAL), Institut de la Nutrition, de l’Alimentation et des Technologies Agro Alimentaires (INATAA), Université Frères Mentouri Constantine 1 (UFMC1), Constantine 25017, Algeria; k_inata@yahoo.fr; 3 Department of Sciences, University of Naples Federico II, 80126 Napoli, Italy; immacolata.speciale@unina.it; 4 Department of Agricultural Sciences, University of Naples Federico II, 80055 Portici, Italy; decastro@unina.it; 5 Group Functionality and Ecology of Beneficial Microbes, Instituto de Investigación Sanitaria del Principado de Asturias (ISPA), 33011 Oviedo, Asturias, Spain

**Keywords:** exopolysaccharide, dairy fermented product, lactic acid bacteria, permeability, viscosity, microstructure

## Abstract

The exopolysaccharide (EPS)-producing *Lactobacillus plantarum* (renamed as *Lactiplantibacillus plantarum*) LBIO1, LBIO14 and LBIO28 strains, isolated from fermented dairy products typical from Algeria, were characterized to evaluate the impact of the polymers in milk fermentations. Their genomes revealed the presence of two complete *eps* clusters of the four described for the reference strain WCFS1. Besides, the three strains presented identical sequences of *eps3* and *eps4* clusters, but LBIO1 and LBIO28 harbour three genes belonging to *eps2* which are absent in the LBIO14 genome. The EPS purified from fermented skim-milks manufactured with the strains showed identical nuclear magnetic resonance (^1^H-NMR) and size exclusion chromatography coupled with a multiangle laser light scattering detector (SEC-MALLS) profiles for polymers LBIO1 and LBIO28, whereas LBIO14 EPS was different due to the lack of the high-molecular weight (HMW)-EPS and the absence of specific monosaccharide’s peaks in the anomeric region of its proton NMR spectrum. The presence of the HMW-EPS correlated with optimal sensorial-physical characteristics of the fermented skim-milks (ropy phenotype). Their microstructures, studied by confocal scanning laser microscopy (CSLM), also showed differences in the organization of the casein-network and the distribution of the bacteria inside this matrix. Therefore, the strain LBIO1 can be proposed for the manufacture of dairy products that require high whey retention capability, whereas LBIO28 could be applied to increase the viscosity.

## 1. Introduction

Lactic acid bacteria (LAB) are a key group a microorganisms playing a pivotal role in food fermentation, being responsible for the quality, safety and nutritional value of the final product [1]. Rural communities have maintained in their heritage traditional uses for food preservation, which has driven to the “domestication” and the selection of strains very well adapted to a specific food environment. However, this practice conducted to a microbial biodiversity loss [2]. Therefore, exploring novel strategies to improve the performance of LAB for industrial application is the focus of research [3], although the natural reservoirs of novel bacteria must be also taken into account for the selection of strains targeted for a potential biotechnological exploitation such as food cultures. Indeed, many LAB have been isolated from traditional fermented products worldwide, including dairy foods [4,5].

The selection of the most suitable LAB for dairy manufacturing involves the search for a series of functional characteristics, among others, the synthesis of exopolysaccharides (EPSs) [6,7,8]. EPSs produced in situ, during milk fermentations, play a pivotal role in the improvement of the sensorial properties of the final product. In fact, they can act as natural bio-thickeners and are efficient viscosifying agents, thus improving the viscosity and texture of low-fat stirred yogurts. Besides, due to their hydrocolloid properties they are effective at retaining water avoiding syneresis in set-type yogurts or increasing the cheese yield [6,7,8]. In addition, these polymers have also attracted interest because they are key molecules linked in the interaction of specific bacteria with the host, thus triggering some of the health benefits induced by the EPS-producing strains [9,10]. Both technological and functional effects on health promotion are due to the extracellular location of EPSs, since they are surface molecules of carbohydrate nature that can be loosely attached to the cell envelope forming a slimy layer, or covalently linked forming a capsule (CPS). In the context of dairy foods, the interaction of the EPS-bacterial layer with the milk matrix, mainly caseins, has a profound impact on the physical-chemical characteristics of the products fermented with EPS-producing LAB [8]. Depending on the chemical composition and the way of synthesis, two main EPS groups, homopolysaccharides and heteropolysaccharides, are distinguishable. The homopolysaccharides are polymerized by means of glycosyl hydrolases (GH) of families GH68 (fructansucrases) and GH70 (glucansucrases) which render β-fructans and α-glucans, respectively [11]. β-linked glucans are intracellularly synthesized by means of a glucosyltransferase which has several membrane segments that facilitate the homopolymer export [12]. The heteropolysaccharides are built from repeating units of monosaccharides (mainly, D-glucose, D-galactose and L-rhamnose) that can be decorated with other sugar and non-sugar monomers. Their synthesis requires clusters of *eps* (or *cps*) genes which, in the case of LAB-EPSs, have a conserved structural organization [9,13]. The chemical composition, size and structure of the EPS molecules are directly linked with their biological functions and technological properties. Generalizing, polymers having negative charge and/or high molecular weight (HMW) are able to modify the viscosity and texture of dairy products [14], or to modulate the immune response in the host [9]. In this regard, the production of a HMW-EPS has been associated with the occurrence of a ropy phenotype in the producing bacterium [15]. This phenotype is denoted by the formation of a long filament when the bacterial colony growing in the surface of an agar-plate is touched with an inoculation loop or, similarly, when a liquid culture is poured forming a strand that remains for some time. The ropy strains are linked to the manufacture of fermented milks with optimal sensorial characteristics, which is dependent on the degree of ropiness desired in the final product [16].

In a previous work, a collection of EPS-producing *Lactobacillus plantarum* (recently reclassified as *Lactiplantibacillus plantarum*, [17]) strains were isolated from traditional Algerian dairy products, identified and preliminarily characterized [18]. In the current work, three of these strains were further studied in order to find their potential application in controlled milk fermentations. To this end, the in silico analysis of their genomes, together with the characterization of the purified EPSs, gave us some clues to understand the physical properties measured in fermented skim-milks manufactured with these strains.

## 2. Material and Methods

### 2.1. Bacterial Strains

The strains *L. plantarum* LBIO1, LBIO14 and LBIO28 were isolated from traditional Algerian dairy foods and were selected for this study based on their ropy character, present in LBIO1 and LBIO28 and absent in LBIO14 [18]. In order to obtain standardized cultures, stocks stored at −80 °C were streaked on the surface of agar-MRS (de Man, Rogosa y Sharpe, Biokar Diagnostics, Beauvais, France) and incubated under aerobic conditions (at 30 °C for 48 h). Afterwards, a single colony was picked up to inoculate 10 mL MRS broth, which was cultivated overnight and used to inoculate (2%) appropriate volumes of fresh MRS that were further incubated for 22 ± 2 h under the same conditions before using them for the different experimental procedures.

For cryo-scanning electron microscopy (SEM), standardized cultures were centrifuged (7780× *g*, 10 min) and 10 times concentrated in Ringer ¼ solution (Merck, Darmstadt, Germany). Finally, these bacterial suspensions were quickly frozen by immersion in liquid N_2_ and sent into dry-ice for microscopic analysis. These bacterial suspensions were visualized by cryo-SEM at the “Electron Microscopy Service” of the Institute of Marine Sciences (ICM-SCIS, Barcelona, Spain). Samples were placed on a cryo-stub, immediately plunged into liquid nitrogen and transferred to the cryo-preparation chamber Quorum PP3000T (Quorum Technologies, Sussex, UK), attached to the microscope. The frozen samples were fractured, sublimed at −90 °C for 5 min, sputter coated with Pt for 30 s and transferred to the Hitachi S-3500N (Hitachi High-Technologies Corporation, Tokyo, Japan) SEM. Observation of samples, kept at −135 ºC, was made at an acceleration voltage of 4 kV.

### 2.2. Genome Analysis

For genome sequencing analysis, total DNA was isolated from overnight grown cultures prepared as previously described. Then cells from 5 mL of each culture were collected by centrifugation (12,070× *g*, 4 °C, 10 min) and DNA was isolated by using the DNeasy blood and tissue kit (Qiagen, Hilden, Germany). Genome sequencing was performed using 250–290 paired-end libraries in a MiSeq instrument (Illumina, San Diego, CA, USA) at GenProbio SRL (Parma, Italy). Genome assemblies were conducted with the PATRIC 3.5.23 online resource (https://www.patricbrc.org/app/Assembly, accessed on June 2019) [19] by using the SPAdes assembler (v. 3.10.0) [20]. Automatic annotation of the open reading frames (ORF) were conducted with RAST (Rapid Annotation using Subsystem Technology) [21] and the NCBI (National Center for Biotechnology Information) Prokaryotic Genome Annotation Pipeline [22]. Deduced nucleotide and protein sequences of interest within the assembled and annotated genomes were individually located through BLASTP (Basic Local Alignment Search Tool Protein homology searches employing, as query, the corresponding protein sequences from the clusters *cps1*, *cps2*, *cps3* and *cps4* annotated in the reference genome *L. plantarum* WCFS1 [23]. Similar approach was used to search for genes of GH68 and GH70 enzymes described from lactobacilli sequences in the NCIB gene database. Besides, carbohydrate metabolic enzymes of the genomes were also annotated through the dbcan tool [24] and comparison against the CAZyme database (http://www.cazy.org accessed on March 2020). RAST annotation was surveyed to search for vitamins biosynthetic pathways. Bacteriocin encoding clusters were screened for with the BAGEL4 web tool [25]. Antibiotic resistance determinants were predicted through homology searches against CARD (Comprehensive Antibiotic Resistance Database) (http://arpcard.mcmaster.ca/ accessed on March 2020) [26] and the RESfinder online tool was used to predict acquired antimicrobial resistance genes or point mutations likely conferring antibiotic resistances [27]. To identify plasmid-associated sequences, raw sequencing reads were analyzed using PLACNETw, a graph-based tool to reconstruct plasmids from next generation sequence pair-end datasets, via the creation of a network of contig interactions [28]. Genomic assembled data were submitted to the GenBank database, under the accession numbers SAMN14671341, SAMN14671394 and SAMN14671401.

### 2.3. Milk Fermentations

Skimmed milk (Difco, Thermo-Fisher Scientific, Madrid, Spain) was reconstituted (11% *w/v*) and supplemented with 1% (*w/w*) pancreatic casein hydrolysate (Sigma, St. Louis, MO, USA) to obtain SMC (skimmed milk-casein) which was pasteurized as previously described [18]. Variable volumes of pasteurized SMC were inoculated (5%) with the standardized MRS cultures previously washed twice with phosphate-buffered saline (PBS). Fermentations were carried out in a thermostatic bath (Julabo TW20 Gmbh, Seelbach, Germany) at 30 °C for variable times, as specified in each section.

### 2.4. Exopolysaccharide (EPS) Purification and Analysis

The EPSs of milks fermented for 48 h were isolated following a procedure previously reported [29]. In a first step, 100 g of fermented milk were mixed with trichloroacetic acid (TCA, 12% final concentration), vigorously stirred for 45 min at room temperature and centrifuged (15,880× *g*, 4 °C, 30 min). The pH of the supernatant was raised to 5.0 ± 0.5 and dialyzed, for 3 days against ultrapure water (dialysis tubes 12–14 kDa MWCO, Sigma, St. Louis, MO, USA), before being freeze-dried. In a second step, 5 mg/mL of the EPS lyophilised powder was dissolved in a buffer containing DNAse type I (2.5 μg/mL final concentration, Sigma, St. Louis, MO, USA) and incubated at 37 °C for 6 h. Later, pronase E from *Streptomyces griseus* (50 μg/mL final concentration, Sigma, St. Louis, MO, USA) was added and the mixture incubated at 37 °C for 18 h. Finally, the same first procedure (starting from the TCA peptide precipitation) was followed to get the purified EPS fraction. The protein content of this fraction was determined by the BCA protein assay protocol (Pierce, Rockford, IL, USA).

The molar mass distribution of the EPS was analysed by size exclusion chromatography (SEC) coupled with a multiangle laser light scattering detector (MALLS; Dawn Heleos II, Wyatt Europe GmbH, Dembach, Germany) as previously described [18]. In short, samples dissolved at 5 mg/mL in 0.1 M NaNO_3_ were separated in two TSK-Gel columns (G3000 PWXL + G5000 PWXL), protected with TSK-Gel guard column (Supelco-Sigma, St. Louis, MO, USA), at 40 °C and 0.45 mL/min flow rate. In addition to MALLS detector for average molecular weight (M_w_) and radius of gyration (R_w_) determination, a PDA (photodiode array) 996 detector (set at 280 nm) and a RI (refractive index) 2414 detector (Waters, Milford, MA, USA) were used to check the presence of proteins and to quantify the amount (μg) of EPS-peaks (using dextran standards for calibration), respectively. The relative abundance of each peak was calculated with respect to the total sum of the peaks.

Proton nuclear magnetic resonance (NMR) spectra were recorded on a Bruker DRX-600 MHz spectrometer (Bruker, Mannheim, Germany) equipped with a cryogenic probe at 298 K. Samples (2–3 mg) were solved in D_2_O (500 μL), spectra were acquired with 16 scans each, 16 K points in resolution, and calibrated on the signal of the residual water signal, set at 4.7 ppm. Standard Bruker Topspin (Topspin 3.5, Bruker) program was used to process the data.

The chemical composition of the polymers was determined analysing the acetylated methyl glycosides (AMG) by gas chromatography mass spectrometry (GC-MS) obtained as follows. To favour EPS dephosphorylation, samples (0.5 mg) were dissolved in 50 μL of 50% aqueous hydrofluoric acid (HF), for 6 h at 25 °C. Then, they were dried in a stream of air and washed with water and methanol, before carrying out their derivatization as peracetylated O-methyl glycosides [30]. Derivatives were then analyzed on Agilent Technologies (Santa Clara, CA, USA) gas chromatograph 7872A coupled with a mass selective detector 5977B and equipped with a Zebron ZB-5 capillary column (Phenomenex, Torrance, CA, USA, 30 m × 0.25 mm internal diameter × 0.25 μm, flow rate 1 mL/min, He as carrier gas). Electron impact mass spectra were recorded with an ionization energy of 70 eV and an ionizing current of 0.2 mA. The following temperature program was employed: 150 °C for 5 min, 150 to 280 °C at 3 °C/min, and 300 °C for 5 min.

### 2.5. Viscosity of Fermented Milks

The dynamic viscosity (η) of SMC fermented until pH 4.5 ± 0.1 (around 24 h) with three strains LBIO1, LBIO14 and LBIO28 was measured at 25 °C using a rheoviscosimeter Haake VT550 (Haake™ Thermo Fisher Scientific, Dreieich, Germany). For that, 10 g of fermented milks samples were carefully placed between the cone and plate (with angle 0.5°) and sheared at shear rate sweeps from 0.1 to 1000 s^−1^ in 180 s, under controlled temperature (25 °C) by the Haake K115 bath connected to the viscometer. The collected data were analysed with the VT1.x0s 550 software (Haake). The flow behaviour of the SMC-fermented samples was described by the Ostwald-de Waele model (power law; equation τ = *Kγ* ^n^, where τ is the shear stress (Pa), K is the consistency index (Pa s^n^), γ is the shear rate (s^−1^), and *n* is the dimensionless flow behaviour index). SMC fermentations were carried out in triplicate for each strain to determine viscosity values.

### 2.6. Macrostructure of Fermented Milks

The permeability (B_t_) of the fermented SMC gels, i.e., the measurement of the flux rate of the aqueous phase (whey) through the casein matrix (milk-gel) giving an indication of the porosity of the fermented milk, was determined as previously reported [31]. As a first step, 4 L (per strain) of fermented SMC (at 30 °C, for 24, to reach pH 4.5 ± 0.1) were manufactured for obtaining the whey by means of centrifugation (15.880× *g*, 30 min, 5 °C) and filtration through filter paper (Whatman filter paper for technical use, Grade 1574 ½, Sigma, St. Louis, MO, USA). The kinematic viscosity (ν) of the whey was measured at 20 °C in a capillary Ubbelodhe type 0C (SI Analytics GmbH, Mainz, Germany). In addition, 1 L (per strain) of pasteurised SMC was inoculated (5%) with strains LBIO1, LBIO14 or LBIO28 and 12 graduated glass-tubes (25 cm length, graduation mark 1 mm, internal diameter 4 mm; Pobel, Barcelona, Spain) open in both ends, were carefully introduced into the inoculated milk. The flasks, with tubes (filled with about 11 cm inoculated milk) inside, were incubated at 30 °C for 24 h. Afterwards, gel-tubes were cleaned and placed in a rack, together with 3 empty reference-tubes, which was introduced in a transparent vat filled with the fermented whey corresponding to each strain. The permeability measurements were performed at 20 °C. With intervals of 1 h (along 8 h) the increase in the level of whey in each tube was annotated and the B_t_ for each tube was calculated using the corresponding formula [31,32]. Finally, the B_t_ of a given fermented milk was calculated from the average values measured in all tubes. This experimental procedure was carried out, at least, two times per strain measuring, at least, 8 tubes in each replicate. 

To determine the capability of the fermented milk gels to retain the aqueous phase, the amount of whey released after applying differential centrifugal forces was quantified [31]. For that, 200 mL of pasteurized SMC were inoculated (5%) with the standardized (PBS-washed) cultures and divided in 4 tubes of 50 mL each, which were incubated in the water bath at 30 °C until pH 4.5 ± 0.1 (24 h). Fermentations were carried out in triplicate for each strain. Afterwards, the tubes were centrifuged at 4 °C for 10 min at four different speeds ranging from 1430 to 15,880× *g* in a fixed-angle conical tube rotor. The quantity of whey released, expressed as a percentage, was calculated by the ratio between the weight of whey recovered and the weight of the initial fermented milk sample.

### 2.7. Microstructure of Fermented Milks

The microscopic structure of milks fermented with the three strains was visualized using confocal scanning laser microscopy (CSLM) and fluorescent dyes [16,31]. For that, the SMC was centrifuged (10,160× *g*, 30 min, 5 °C) to remove most non-dissolved milk particles before pasteurization. Then, after pasteurization, two dyes were added: rhodamine B (Sigma, St. Louis, MO, USA) and acridine orange (Sigma) at final concentrations of 0.001% (*w/v*) and 0.002%, (*w/v*) respectively. Rhodamine B dyes proteins and acridine orange bacterial nucleic acids. After proper homogenisation, stained milk was inoculated (5%) with the PBS-washed MRS cultures and 2 mL were carefully placed into high-optical quality plastic 2-wells μ-slides (Ibidi GmbH, Gräfelfing, Germany) for direct CSLM analysis. The μ-slides were incubated at 30 °C for 24 h (to a pH about 4.5) to ensure the formation of the milk-gel. The confocal microscope Ultra-Spectral Leica TCS AOBS SP2 (Leica Microsystems GmbH, Wetzlar, Germany) located in the University of Oviedo facilities (Oviedo, Asturias, Spain) was used. Bacteria dyed with acridine orange were visualized with the laser 488 nm ion argon/krypton (green), and proteins (mainly caseins) dyed with rhodamine B were visualized with the laser 543 nm nm He/Ne (red), but also with the laser 488 nm. Thus, after image treatment with the program LCS (Leica Microsystems GmbH, Wetzlar, Germany), bacteria were visualized in green and the casein matrix in yellow (combination red and green). Z-stack images were obtained using a 63×/1.40NA oil objective (1.58 zoom) and the 3D reconstruction was performed using the Leica LCS and the Confocal Uniovi Image J software.

### 2.8. Statistical Analysis

The IBM-SPSS statistics for Window version 25.0 (IBM Corp., Armonk NY, USA) was used to analyse the quantitative data. One-way analysis of variance (ANOVA) and the SNK (Student–Newman–Keuls) mean comparison tests were used to assess the differences (*p* < 0.05) among the three strains used in this study.

## 3. Results and Discussion

### 3.1. Insight into the Genomes of L. Plantarum Strains: Focus on eps Cluster Analysis

**Figure 1:**
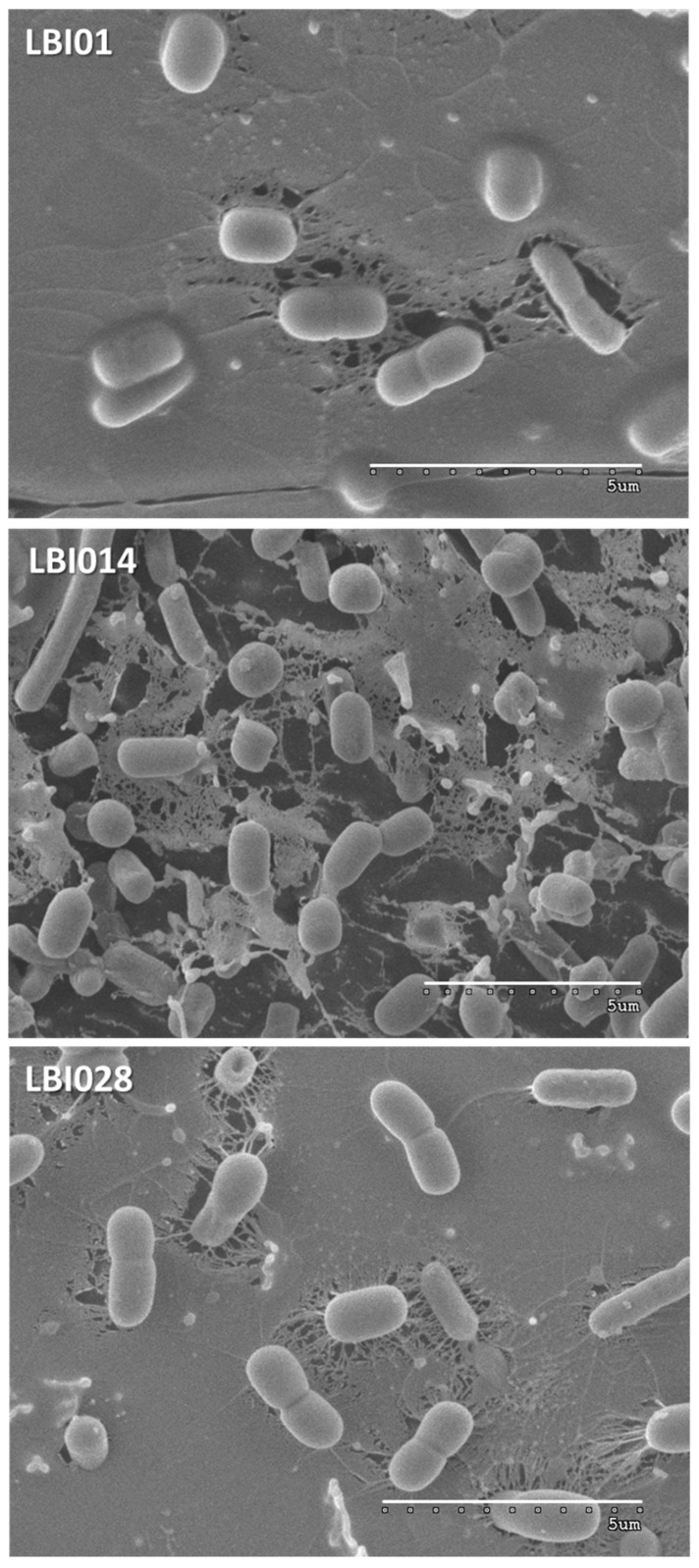
Cryo-scanning electron microscopy (cryo-SEM) of bacterial biomass collected from 24 h MRS broth cultures of the exopolysaccharide (EPS)-producing *L. plantarum* strains LBIO1, LBIO14 and LBIO28. The EPSs are the matrix surrounding the bacterial surface. Bar 5 μm.

The *L. plantarum* strains used in this study had different origins, as they were isolated from different dairy fermented products manufactured from cow milk in different geographical areas. The strain LBIO1 was isolated from the soft-ripened cheese “Bouhezza” and LBIO14 from the fresh cheese “Klila” both in the region of Batna, whereas LBIO28 was obtained from the fermented milk “Rayeb” in the region of Bordj El Ghadir [5,18]. The strains were selected based on the presence (LBIO1 and LBIO28) or absence (LBIO14) of the ropy character [33]. Since the ropy character is directly linked to the synthesis of certain type of EPS, our first approach to characterize these strains was the visualization of the bacterial surface using cryo-SEM. This is one of the less destructive electron microscopy techniques that only involve freezing the sample in liquid N_2_ and is thus, highly recommended to avoid changes on EPS structure induced by dehydration of samples [34]. Surprisingly, the three lactobacilli strains presented an EPS-like structure around their surface (Figure 1), but that of the ropy LBIO1 and LBIO28 strains was much more dense and compact than observed for the non-ropy LBIO14. This suggests that the last one produces less polymer and/or produces an EPS having different length (molecular weight), whereas the ropy strains produced a slimy polymer able to establish a compact network entrapping and connecting inside several cells. As far as we could find, there are no reports in literature regarding visualization of EPS-producing LAB under cryo-SEM. Nevertheless, the structure observed for LBIO1 and LBIO28 EPSs resembles that found with the same technique for ropy EPS-producing bifidobacteria [10]. The use of SEM for the visualization of some EPS-producing lactobacilli, which involves different fixation steps prior to coating, gives similar homogeneous sheet-like structures [35]. However, more open-loose (porous) structures, such as the ones detected for LBIO14 EPS, were also reported [36]. In our study, the three EPS-producing lactobacilli were not submitted to dehydration, so the structure differences detected among the strains must be due to intrinsic differences of their polymers.

In a further bacterial characterization, the genetic fingerprinting obtained by RAPD-PCR amplification showed that only the primer M13, a probe designed from the bacteriophage M13 of *Escherichia coli*, allowed the identification of distinctive band patterns among the three strains (see results and methodology details for this analysis in Supplementary Figure S1), as previously reported for other LAB [37]. This likely suggests that the *L. plantarum* isolates under study represent different strains. In order to better characterize their genetic background, the genomes of LBIO1, LBIO14 and LBIO28 were sequenced and their sequences held in the NCIB GeneBank database (Table 1). 

Overall, the three draft genomes were 3.19, 3.15 and 3.18 Mbp length, respectively, an average GC content ranging from 44.32 % (LBIO1 and LBIO28) to 44.44 % (LBIO14), and a total of 3113, 3072 and 3108 ORFs which could be predicted and annotated. These general values are in agreement with the available data for other reported *L. plantarum* genomes [38,39]. Some genome-encoded potential traits of these strains, that could provide additional functional (health promoting) or technological benefits for their application into the elaboration of fermented milks, were screened through RAST annotation and in silico homology searches. For instance, the genomes of the three strains included genes involved in the pathways for riboflavin, folate and pyridoxin biosynthesis, while the machinery required to synthesize other vitamins, such as thiamine and biotin, are incomplete, as previously described in other *L. plantarum* strains. Indeed, the vitamin-producing strains could help produce fortified fermented foods, aiding to prevent nutritional deficiencies in target populations [23,39]. On the other hand, a search against the BAGEL4 database identified putative bacteriocin encoding gene clusters in the three strains, trait that was previously reported for others and could have potential applications to extend the product shelf-life, conferring protection against spoilage and foodborne pathogens [40]. In relation to genetic encoded traits which may impede the technological exploitation of microbial strains, such as the presence of antibiotic resistance genes or virulence factors, it is worth remarking that no potential antibiotic resistance or virulence determinants were detected in any of the three genomes, accordingly with results from other strains of the same species [39].

**Figure 2:**
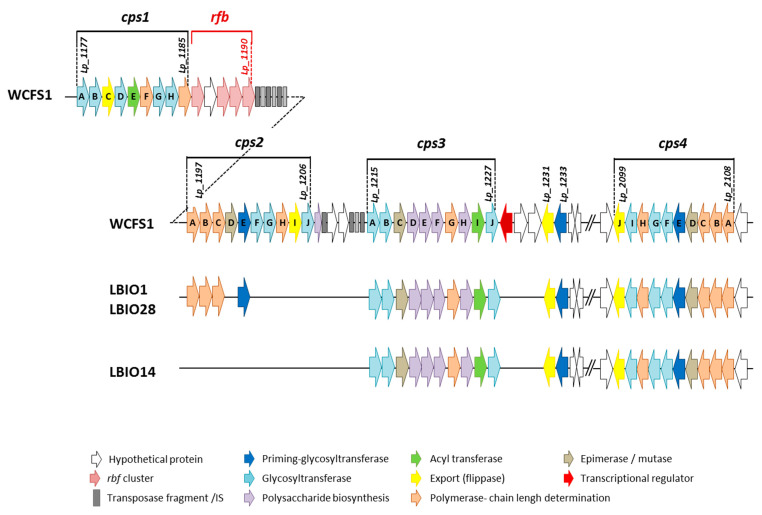
Physical maps of the biosynthetic *eps* clusters found in the genome (chromosome) of the strains *L. plantarum* LBIO1, LBIO14 and LBIO28 in comparison with the reference strain WCFS1. The length of the arrow is not proportional to the length of the predicted open reading frame (ORF). For detailed description of the genes and percentages of homology, see Supplementary file 1.

In agreement with the aim of this work, the genome analysis of these strains was focused to the genes involved in the EPS-synthesis. None of the approaches applied to search for genes involved in the production of β-fructans and α-glucans revealed any significant hit, suggesting that the three genomes did not harbor genes coding for GH68 and GH70 enzymes. The production of these type of homopolysaccharides was demonstrated only for very few *L. plantarum* strains, such as the α-glucans synthesized by the strains DM5 [41] and CIDCA 8327 [42]. A closer look at the genetic determinants linked to the synthesis of heteropolymer-like EPSs revealed the presence of two complete *eps* clusters (*eps*/*cps3* and *cps4*), of the four described for the strain *L. plantarum* WCFS1 [43], in the three strains of this study (Figure 2, Supplementary file 1). The *cps1* cluster was absent, whereas three (*cps2ABC*) out of the 10 genes belonging the WCFS1 *cps2A-J* cluster were detected only in the ropy LBIO1 and LBIO28 strains. The gene *cps2E* (in WCFS1) encoding a priming-glycosyltransferase (p-GTF) showed a very low degree of homology (40%, Supplementary file 1), thus we consider that was not present in LBIO1 and LBIO28 clusters. 

The absence of these three *cps2* genes in LBIO14 was the only difference found among the three Algerian origin lactobacilli, since the homology (% of genetic identity) of these genetic regions was 100% among them). Therefore, taking into account the pattern obtained from the comparison of 43 *L. plantarum* genomes [44], the LBIO1 and LBIO28 strains fit in the type B pattern (complete eps/*cps3* and *cps4* and partial *cps2*) whereas LBIO14 fit into type C pattern (two clusters, in this case *cps3* and *cps4*). Apparently, the *eps* clusters from our strains are not harboured in plasmids, as suggested using the in silico PLACNET search network analysis tool, which revealed that the *eps* genes are not included in contigs displaying homology to plasmid replication proteins. The homology of our *eps* genes in comparison with those of WCFS1 strain was, in general, higher than 94%. Only the GTF *cps3A* presented lower homologies, around 70%, in our strains (Supplementary file 1). The *cps4* cluster seems to be present in all *L. plantarum* strains currently analyzed and is located in a chromosomal region separated from that containing the other three clusters, as is the case in our genomes. However, *cps1* seems to be present in only a few strains [44]. As previously described by Remus and co-workers in WCFS1 strain [43], *cps2A-J* and *cps4A-J* have the complete “consensus” functional structure required for the synthesis of EPS. That is, both harbour genetic elements involved in polymerization/ chain-length determination, a core of GTFs starting with the p-GTF, and genes related to polymerisation/(flippase-type) export, all flanked with mobile elements and keeping the same gene order and orientation within the *eps*-encoding clusters [9]. In general, the p-GTF initiates the intracellular synthesis of the EPS repeating units after being linked to a lipid membrane carrier. Latter the GTFs catalyzes the addition of different monosaccharides to form the repeat unit which is translocated across the membrane by means of a flippase-system. Finally, the polymerization of the repeating units takes place extracellularly by means of a polymerase, and other proteins are involved in the determination of the chain length of the polymer. The presence of transposases and insertion sequences flanking *eps* clusters is a common characteristic among bacteria, which would facilitate the horizontal transfer of *eps* genes among different bacteria that occupy a common habitat [9]. Genes involved in regulation seemed no to be present inside the *cps* loci coding for EPS biosynthesis in *L. plantarum* WFS1 [43], as is the case for other LAB [13,45]. The WCFS1 *cps1* and *cps3* clusters lack some key-genes for EPS production, such as the p-GTF that initiates the intracellular synthesis of the repeating units. However, these clusters were required for the synthesis of the capsular EPS in this strain, as it was demonstrated by the construction of several gene-deletion and cluster-deletion mutants [43]. The EPS produced by the mutant Δ*cps1A-J* genes has modified the molecular weight and lacks rhamnose, whereas mutations in the other three individual cluster deletions, or the quadruple-deletion, resulted in a reduction of the amount of polymer synthesised. Later, by means of transcriptional analysis it was found that the four *cps* loci of WCFS1 were organized in five operons, three of them under the control of the global regulator CcpA [46]. It is worth noting that *L. plantarum* WCFS1 does not have a ropy phenotype, which was present in the strains SF2A35B and Lp90 [47]. The genomic comparison among these strains revealed that Lp90 harbors the four *cps* clusters and SF2A35B lacks *cps1*, as is the case of the ropy LBIO1 and LBIO28 reported here. Moreover, *cps2* seemed to be the most variable among WCFS1, SF2A35B and Lp90 strains since additional (not orthologs) genes are present in the last two ropy-strains [47]. These authors conclude that the set of genes present in SF2A35B and Lp90 *cps2* is involved in the occurrence of the ropy phenotype since their deletion causes the loss of this character. In our case, the differences between the ropy LBIO1 and LBIO28 *L. plantarum* strains and the non-ropy LBIO14 were also located in *cps2*, which was not present in the last one. However, in our case LBIO1 and LBIO28 *cps2* had a reduced (not increased) number of genes with respect to WCFS1. Nevertheless, the three genes present in these strains are related with the polymerization and chain length regulation (see Supplementary file 1). This finding suggests that the *cps2ABC* found in LBIO1 and LBIO28 might play a relevant role in the synthesis of the HMW-EPS responsible for the synthesis of a ropy polymer. 

### 3.2. Characterization of EPS Purified from Fermented Milks

To determine whether differences among the three polymers could be detected in milk products fermented with either of the strains under study, the purified EPS fractions obtained from milks fermented with the three *L. plantarum* strains were analysed by means of SEC-MALLS. The SEC profile shows the presence of three major peaks differing in size, showing average retention times about 27, 38 and 42 min. The ropy LBIO1 and LBIO28 EPSs presented the three peaks whereas the non-ropy LBIO14 lacks that corresponding with the biggest polymer fraction (Figure S2; [18]). It is worth noting that in the smallest peak (42 min) the ultraviolet (UV)-detector signal (set at 280 nm) was very intense in the three purified EPS, with denotes the presence of protein in all the polymer fractions. This was supported by the results obtained with the BCA-protein assay that showed a protein content of 31.8, 98.5 and 38.9 μg/mg for the EPS fractions purified from LBIO1, LBIO14 and LBIO28 fermented milks, respectively. Given that in this work an additional treatment with DNAse and pronase E, followed by 12%-TCA precipitation, was performed for EPS purification, this suggests that proteins could be strongly linked to the surface structures extracted. In this regard, some authors have reported that proteins could form part of cell-wall polysaccharides [48], although other surface structures (such as teichoic acids) could have co-precipitated with the polysaccharides [49,50]. Nevertheless, most authors do not report checking the presence of proteins in the EPS they have characterized. In our EPS fractions, the peak 3 has an average molecular weight (M_w_) of 4 to 9 kDa, depending on the strain, and it is the most abundant in the three EPS fractions (Table 2). Thus, we cannot discard the presence of a smaller EPS fraction in this peak [51,52]. As indicated above, the most noticeable finding in the SEC-MALLS analysis was the presence of the high M_w_ peak (about 1.2 × 10^6^ Da) in LBIO1 and LBIO28 strains, with a relative abundance of 16% in both cases. The presence of this high-molecular weight (HMW) peak could explain the ropy character of these strains [15,53], whereas its absence could explain the non-ropy phenotype in the LBIO14 strain. 

**Figure 3:**
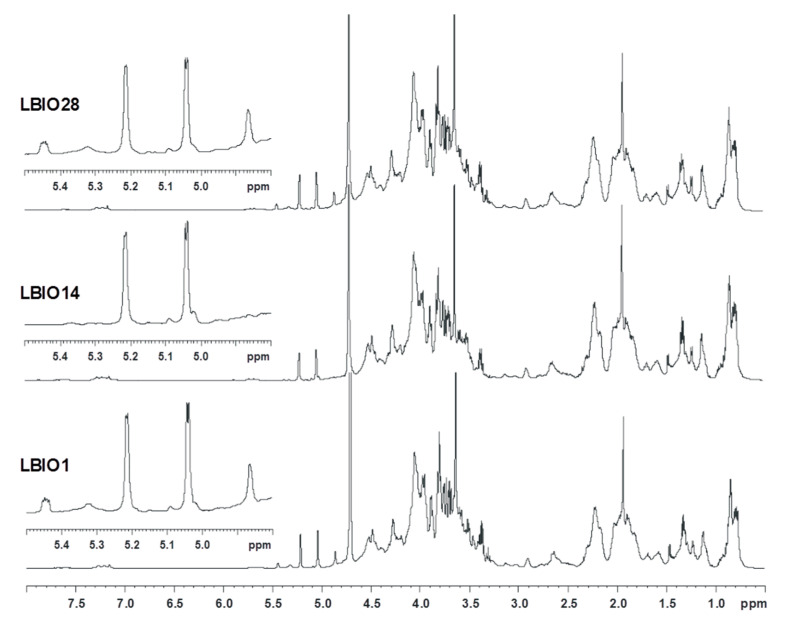
Proton spectra measured by nuclear magnetic resonance (+H-NMR) for the EPSs purified from milks fermented with *L. plantarum* LBIO1, LBIO14 or LBIO28. The anomeric region corresponding with EPSs is enlarged in the left corner of the spectra (amplified range 5.5–4.8 ppm).

The purified EPS fractions were also examined via proton NMR (Figure 3). The three spectra profiles were rather similar and denoted that the carbohydrate-related material was co-extracted with lipid-like substances and/or proteins resistant to the purification treatment, as inferred from the signals at 3.0–0.5 ppm. As for the carbohydrate component, the three spectra had a crowded anomeric region, with signals representative of monosaccharide residues both α (5.5–4.8 ppm) and β (4.65–4.3 ppm) configured to the anomeric centre. Moreover, some differences were noted in the 5.5–4.8 ppm region, where all samples shared two main signals at 5.21 and 5.04 ppm, representative of α-configured monosaccharides, in agreement also with their small coupling constant value (^3^J_H1H2_ 2.95 and 3.41 Hz, respectively). However, the two ropy strains, LBIO1 and LBIO28, had two additional peaks at 5.44 and 4.86 ppm, of lower intensity, which seem to be related to the EPS, as they are absent in LBIO14, the non-ropy strain.

This finding was also confirmed by preliminary data performed on the same strains collected from the biomass obtained from agar-MRS grown plates. Regarding the structure of the EPSs, their repeating unit likely presents a phosphodiester linkage, as suggested by the peculiar shape of the signal at 5.44 ppm that is representative of a sugar in the α-configuration attached to a phosphate group. This finding is not unusual since there are several cases in LAB strains in which these non-carbohydrate substituents are part of EPS/CPS structures [14,54,55] or where two repeating units are joined by a phosphodiester linkage [50].

The monosaccharide analysis of the LBIO1, LBIO14 and LBIO28 polymers obtained from fermented milks has been performed with dephosphorylated samples to appreciate the presence of teichoic acids. The three samples had glucose as the main component, followed from galactose (about 35 % of the glucose), along with traces of mannose, hexosamines and neuraminic acid (Table 3). The latter has already been found as component of lactobacilli polymers, including *L. plantarum* [56]. Importantly, this analysis disclosed the presence of glycerol and ribitol, the two polyols related to teichoic acid (Supplementary Figure S3). This finding indicated that teichoic acids were co-extracted together with EPS, and also that they were more abundant in LBIO14 (Table 3) compared to LBIO1 and LBIO28 polymers. Accordingly, LBIO1 and LBIO28 have higher proportions of other carbohydrate molecules, the EPS, which we suppose related to the ropy phenotype of the two strains.

A preliminary purification by ion exchange chromatography failed in the isolation of the EPS-related material from the polymeric material obtained from the fermented milks. Thus, purification and structural studies of EPS from LBIO1, LBIO14 and LBIO28 strains will be the object of further work. The physical-chemical properties of EPSs purified from different *L. plantarum* strains have been characterized and all of them presented glucose and galactose in their composition [8,44], such as we have denoted in our polymers. Very often, these monosaccharides are combined with other monomers such as mannose [8], fructose [57], N-acetyl-galactosamine [58], galactosamine, glucosamine [47], rhamnose [50], glucuronic acid [59] and/or glycerol [50,58], among others. The physical-chemical characteristics of the EPS are of special relevance for their functional and technological characteristics [14,60].

### 3.3. Characterization of Skim Milks Fermented with the EPS-producing L. Plantarum

**Figure 4:**
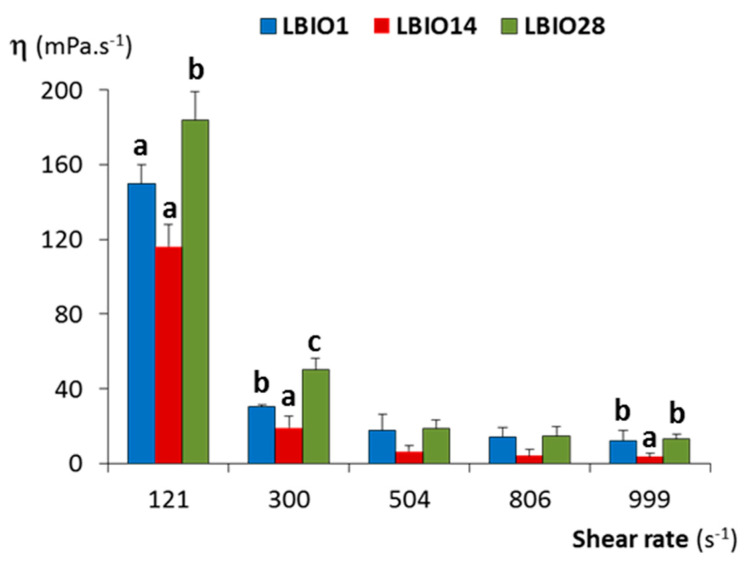
Dynamic viscosity (η) at different shear rates of stirred fermented milks obtained after fermentation for 24 h of skim-milk with the EPS-producing strains *L. plantarum* LBIO1, LBIO14 or LBIO28. Within each shear rate, bars that do not share a common letter are significantly different according to analysis of variance (ANOVA) and Student–Newman–Keuls (SNK) (*p* < 0.05) mean comparison test.

The flow behaviour and viscosity of milks fermented with the three strains were analysed using a rotational viscometer. At low shear rate, the apparent viscosity decreased when increasing the shear rate in all cases (Figure 4). This flow is a typical shear-thinning and non-Newtonian behaviour, characteristic of pseudoplastic fluids, as reported by several authors for milks fermented with EPS-producing and non-producing LAB [61,62]. At higher shear rates, the viscosity decreased and remained without noticeable changes, then behaving as a Newtonian fluid [63].

The flow parameters obtained at shear rate of 300 s^−1^ (Supplementary Table S1) showed that the value of the flow behaviour index “*n*” was lower than 1 for the three fermented milks, which confirmed this shear-dependent behaviour [61]. The “*n*” index, as well as the consistency coefficient “K”, were significantly different (*p* < 0.05) among the three fermented milks; those fermented with the strain LBIO28 presented the higher K and lower n values. In agreement with this, different viscosity was observed among the three fermented milks (*p* < 0.05). LBIO28-fermented milks had values higher than those fermented with LBIO1 or LBIO14 strains and, as it could be expected, the non-ropy one presented the lowest viscosity. This fact is in agreement with the well-known statement that the capability to modify the viscosity of fermented milks is a strain and/or EPS-dependent feature [64]. It seems that LBIO28-fermented milks had a consistent structure that is more difficult to breakdown during shearing which, presumably, could be linked to the degree of ropiness of its EPS [65]. It seems that the role of this polymer is slowing the breakdown of the casein network through establishing strong EPS-protein interactions [66]. At this time, we cannot explain the influence of specific chemical or structural features exclusive of the LBIO28 EPS on viscosity and casein-interactions.

**Figure 5:**
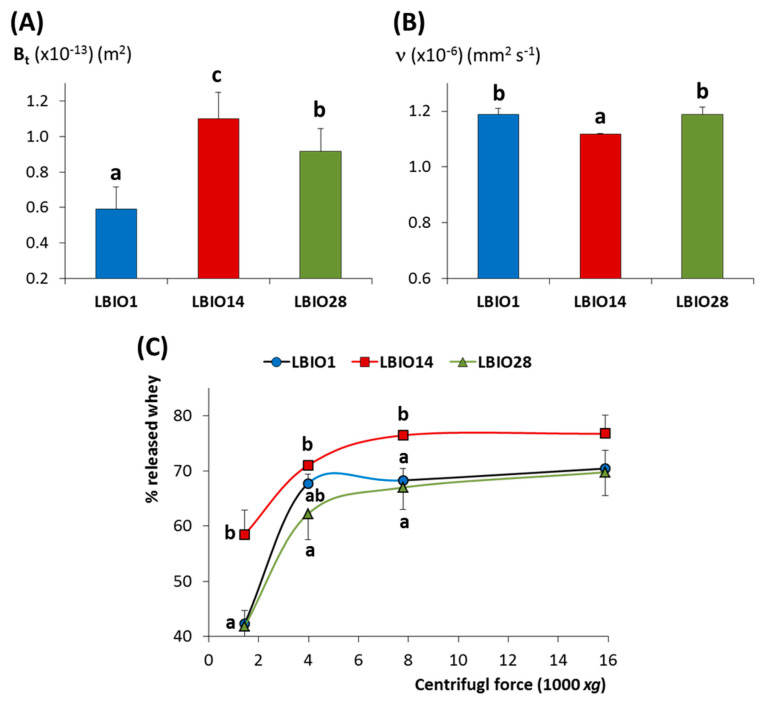
Physical parameters of skim milks fermented with the EPS-producing strains *L. plantarum* LBIO1, LBIO14 or LBIO28. (**A**) Mean permeability coefficient (B_t_) of the fermented milk-gels; (**B**) Kinematic viscosity (ν) obtained by Ubbelohde measurement of the whey used to measure the permeability coefficient. (**C**) Amount of whey separation (% *w/w*) as a function of centrifugal force of fermented milk-gels. Bars or symbols (within each centrifugal force) that do not share a common letter are significantly different according to ANOVA and SNK (*p* < 0.05) mean comparison test.

The structure of a fermented skim-milk gel is formed along the acidification due to the bacterial metabolism of lactose to (mainly) lactic acid, which generates a casein-network that encloses the aqueous (whey components) phase into pores. The flow properties of this matrix is related to the porosity degree of the casein aggregates, as well as to the viscosity of the whey, and it can be determined by measuring the coefficient of permeability over time (B_t_) [67]. The three milk gels fermented with the lactobacilli strains under study showed B_t_ coefficients between 0.6 to 1.1 × 10^−13^ m^2^ (Figure 5A). The B_t_ value obtained for the non-ropy LBIO14 strain (1.10 ± 0.15 × 10^−13^ m^2^) fitted into the range reported for milk gels acidified at 30 °C with glucono-δ-lactone (1–2 × 10^−13^ m^2^; [67]). Whereas, those of milk gels obtained with the ropy-strains were lower (0.59 ± 0.12 and 0.92 ± 0.13 × 10^−13^ m^2^, for LBIO1 and LBIO28 milk-gels, respectively). In fact, statistical differences (*p* < 0.05) were found among the B_t_ coefficients of the three strains (Figure 5A). LBIO14 gels were the most permeable as it was previously reported for gels formed with non-EPS producing LAB strains [31]. This is related with the lowest viscosity of the LBIO14 whey (1.12 ± 0.002 × 10^−6^ mm^2^ s^−1^) that flowed through the casein network, as it was detected by measuring the kinematic viscosity (ν) of the three whey samples obtained from the fermented milks (Figure 5B). The lowest viscosity of the non-ropy LBIO14 whey is linked to the absence of the HMW-EPS fraction. As expected, the ν values obtained for the ropy LBIO1 and LBIO28 whey were higher than for LBIO14, but were equal between them (1.19 ± 0.02 × 10^−6^ mm^2^ s^−1^). This suggests that the porosity of the casein network, or the EPS–casein matrix interactions, accounted for the variations in the B_t_ coefficient detected between these two strains [66]. No significant differences in the capability to retain whey between LBIO1 and LBIO28 milk gels were detected after applying different centrifugal forces (Figure 5C), although these strains were able to hold more whey (*p* < 0.05) than the non-ropy LBIO14 (Figure 5C). This was due to the presence of the HMW-EPS, as previously reported for other EPS-producing LAB [68].

**Figure 6:**
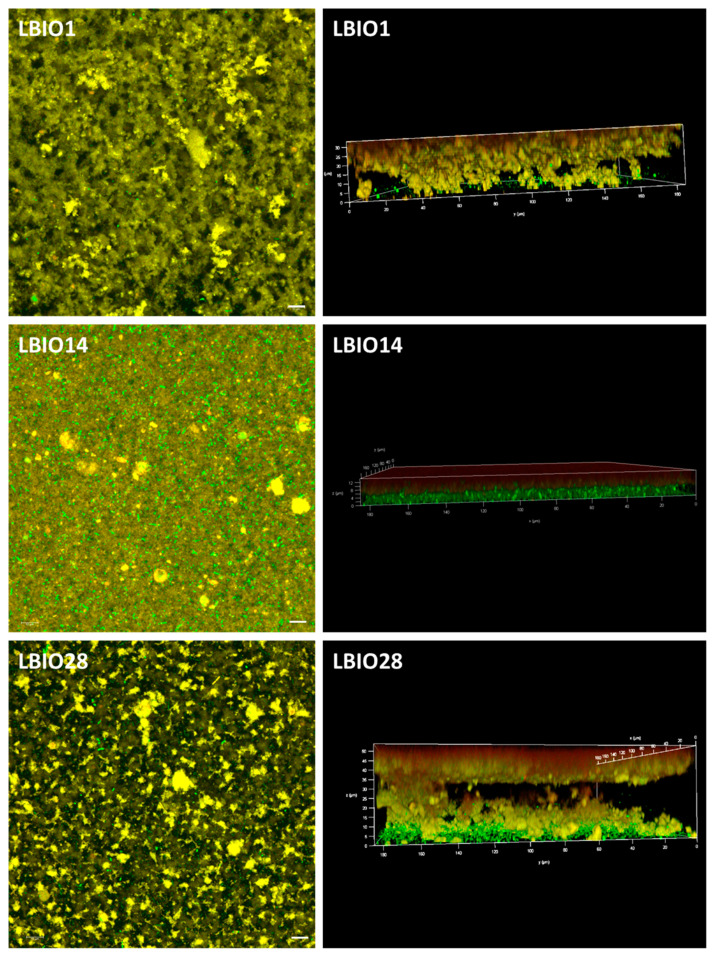
Three-dimensional microstructure of skim-milk gels (casein network stained in yellow) fermented with the EPS-producing strains *L. plantarum* LBIO1, LBIO14 or LBIO28 (stained in green). Left microphotographs (bar 10 μm): 3D projection (visualized in XY axes) of several Z-stack images obtained using a 63×/1.40NA oil objective on an inverted Ultra-Spectral Leica TCS AOBS SP2 confocal microscope. Right microphotographs: rotation of the 3D Z-stack images, to see the projection in the three axes (XYZ).

On this point, it is worth noting that the acidification rate and the increase in the number of bacteria during fermentation were different among the strains (Supplementary Table S2; [18]). The strain LBIO28 was able to drop the pH faster and to accumulate more EPS around the gelation point than LBIO1 [18], both parameters influencing the gel permeability. It seems that the formation of LBIO1 gels, that is the casein aggregates enclosing pores, took place before the highest accumulation of EPS. Then, the polymer surrounding the bacteria mostly occupies the pores, partially blocking the flow through them (reducing permeability). In the case of LBIO28 gels, the acidification and synthesis of the highest amount of polymer runs parallel, thus the EPS tended to be distributed between the casein network and the pores, which were not totally blocked with EPS (higher permeability). Similar findings were previously reported [31,69], and they can be supported with the microstructure of the three fermented milks visualized under CLSM (Figure 6). In fact, the more permeable gel obtained with the non-ropy LBIO14 strain showed a coarse and dense protein network with non-distinguishable pores and the bacteria precipitated in the bottom (green sediment in the 3D-rotated micrograph), because the acidification rate was the slowest (Supplementary Table S2). On the contrary, “black” pores or “void” spaces were detected into the casein-aggregates network of the ropy LBIO1 and LBIO28 gels. In the first type of gel, the pores were more regularly distributed and the casein structure, with the bacteria and their polymers entrapped inside, was more homogeneous than that of LBIO28 gel. In the latter, the EPS more equally distributed into the protein matrix could be linked to caseins, causing a tightening of the protein network that is more resistant to mechanical action. Thus, the permeability and microstructure of the casein aggregates formed during bacterial milk fermentation were depending on the strain used, that determined the acidification rate, as well as the type of polymer produced that might have influenced the interactions EPS-casein network [31,70,71].

## 4. Conclusions

Summarizing, in this work we have found that the three *L. plantarum* strains isolated from traditional Algerian fermented milks have EPS surrounding their surface but showing different structure. This is probably due to the presence of a HMW-EPS detected only in the ropy LBIO1 and LBIO28 strains but absent in the non-ropy LBIO14. Although the genomes of the strains were quite similar, the main difference among the strains, in comparison with *L. plantarum* WCFS1, was denoted in the *cps2* cluster which was only present in the ropy strains. This points to its implication in the synthesis of the HMW-EPS and thereby, in the occurrence of the ropy phenotype. Following, the physical characterization of skim-milks fermented with the three strains allowed us to propose the technological applications of the ropy EPS-producing strains. *L. plantarum* LBIO1 produced milk gels with a less permeable structure, and thus this strain could be used for the manufacture of dairy products to avoid syneresis and/or to increase the water retention. Milks fermented with *L. plantarum* LBIO28 presented the highest viscosity, indicating that the polymer produced in situ can be used as a natural fat replacer to improve the rheological properties of less caloric products.

## Figures and Tables

**Table 1:** Some features of the genomes from the *L. plantarum* strains used in this study. The LBIO1, LBIO14 and LBIO28 genome sequences are available in the GenBank database (http://www.ncbi.nih.gov/genbank).

	Genome					
	Accession No.	Length (bp)	CDSs	%G + C	rRNA	tRNA
**LBIO1** (Ropy)	SAMN14671341	3,186,843	3113	44.32	9	66
**LBIO14** (non-ropy)	SAMN14671394	3,155,118	3072	44.44	10	68
**LBIO28** (Ropy)	SAMN14671401	3,187, 061	3108	44.32	10	66

**Table 2:** Analysis of the average molecular weight (M_w_) and radius of gyration (R_w_) determined by size exclusion chromatography coupled with multiangle laser light scattering (SEC-MALL) of EPSs purified from skim-milks fermented for 48 h with the *L. plantarum* LBIO1, LBIO14 or LBIO28 strains. The Supplementary Figure S2 shows the SEC-MALLS (LS detector 90º) chromatograms.

		Mean ± Standard Deviation (SD)		
Strain	Parameters	Peak 1	Peak 2	Peak 3
**LBIO1** (Ropy)	Retention time (min)	26.83 ± 0.01	37.61 ± 7.40	41.52 ± 0.01
	M_w_ (× 10^3^ g/mol)	1154.5 ± 16.26	19.9 ± 0.07	4.41 ± 0.47
	R_w_ (nm)	74.6 ± 2.26	34.8 ± 11.9	68.9 ± 1.34
	% relative abundance	15.8 ± 0.1	15.3 ± 0.2	68.2 ± 0.2
**LBIO14** (No-ropy)	Retention time (min)	ND	37.80 ± 0.01	41.54 ± 0.01
	M_w_ (× 10^3^ g/mol)		20.3 ± 1.57	8.81 ± 3.61
	R_w_ (nm)		39.0 ± 8.9	137.1 ± 42.0
	% relative abundance		14.6 ± 0.0	85.0 ± 0.1
**LBIO28** (Ropy)	Retention time (min)	26.73 ± 0.01	37.63 ± 0.01	41.53 ± 0.01
	M_w_ (× 10^3^ g/mol)	1183.5 ± 43.13	22.4 ± 0.08	9.90 ± 0.11
	R_w_ (nm)	74.7 ± 0.50	45.9 ± 0.7	143.1 ± 0.56
	% relative abundance	15.9 ± 0.5	15.2 ± 0.2	68.0 ± 0.3

ND: no detected.

**Table 3:** Monomer composition, as ratio referred to glucose or as percentage (in brackets), of acetylated methyl glycosides (AMG), after dephosphorylation with aqueous hydrofluoric acid (HF), of EPSs purified from skim-milks fermented for 48 h with the *L. plantarum* LBIO1, LBIO14 or LBIO28 strains. The Supplementary Figure S3 shows the GC-MS (gas chromatography coupled with mass spectrometry) chromatograms.

	Monomer ^1^ Ratio/(%)							
EPSs	Glycerol	Ribitol	Man	Gal	Glc	GalN	GlcN	NeuA
**LBIO1**	0.06 (3.2)	0.19 (10.1)	0.07 (3.7)	0.38 (20.1)	1.00 (52.9)	0.05 (2.6)	0.07 (3.7)	0.07 (3.7)
**LBIO14**	0.12 (5.4)	0.31 (13.8)	0.14 (6.3)	0.33 (14.7)	1.00 (44.6)	0.09 (4.0)	0.12 (5.4)	0.13 (5.8)
**LBIO28**	0.06 (3.4)	0.16 (8.9)	0.08 (4.5)	0.36 (20.1)	1.00 (55.9)	0.03 (1.7)	0.05 (2.8)	0.05 (2.8)

**^1^** Man: mannose, Gal: galactose, Glc: glucose, GalN: galactosamine, GlcN: glucosamine, NeuA: neuraminic acid.

## Supplementary Materials

The following are available online at https://www.mdpi.com/2076-2607/8/8/1101/s1, **Figure S1**: genetic fingerprinting obtained by polymerase chain reaction (PCR) amplification of different genomic regions from *L. plantarum* LBIO1, LBIO14 and LBIO28 DNA. **Figure S2**: SEC-MALLS chromatograms of the EPS-fraction extracted from milks fermented with the *L. plantarum* LBIO1, LBIO14 and LBIO28 strains. **Table S1**: rheological parameters of milks fermented with *L. plantarum* LBIO1, LBIO14 and LBIO28 strains. **Table S2**: acidification rate and increase of bacterial counts of milks fermented with *L. plantarum* LBIO1, LBIO14 and LBIO28 strains. **Supplementary File 1**: ORF location, gene function, and % of identity of genes included in the *eps* clusters found in LBIO1, LBIO14 and LBIO28 *L. plantarum* genomes in comparison to that of the strain WCFS1.
