# Supplementary material for: Selection of Exopolysaccharide-Producing Lactobacillus Plantarum (Lactiplantibacillus Plantarum) Isolated from Algerian Fermented Foods for the Manufacture of Skim-Milk Fermented Products
Source: Microorganisms. 2020 Jul 23;8(8):1101. doi: 10.3390/microorganisms8081101 (PMC7465087; doi:10.3390/microorganisms8081101)
Supplement: Supplementary file 1 [file microorganisms-08-01101-s001.pdf]

Supplementary Figure S1: Primer used for typing the *L. plantarum* LBIO1, LBIO14 and LBIO28 isolated from Algerian traditional dairy products and electrophoretic band profile obtained.

**Name**	**Nucleotide sequence**	**Reference**
OPA-18	5'- AGGTGACCGT -3'	Roy *et al*., 2000
M13	5'-GAGGGTGGCGGTTCT-3'	Huey & Hall, 1989
ISSRev	5'-GGATCCAAGACAACGTTTCAAA-3'	Acedo-Félix & Pérez-Martínez, 2003
BOX A2R	5'-ACGTGGTTTGAAGAGATTTTCG-3 '	Nayak *et al*., 2011

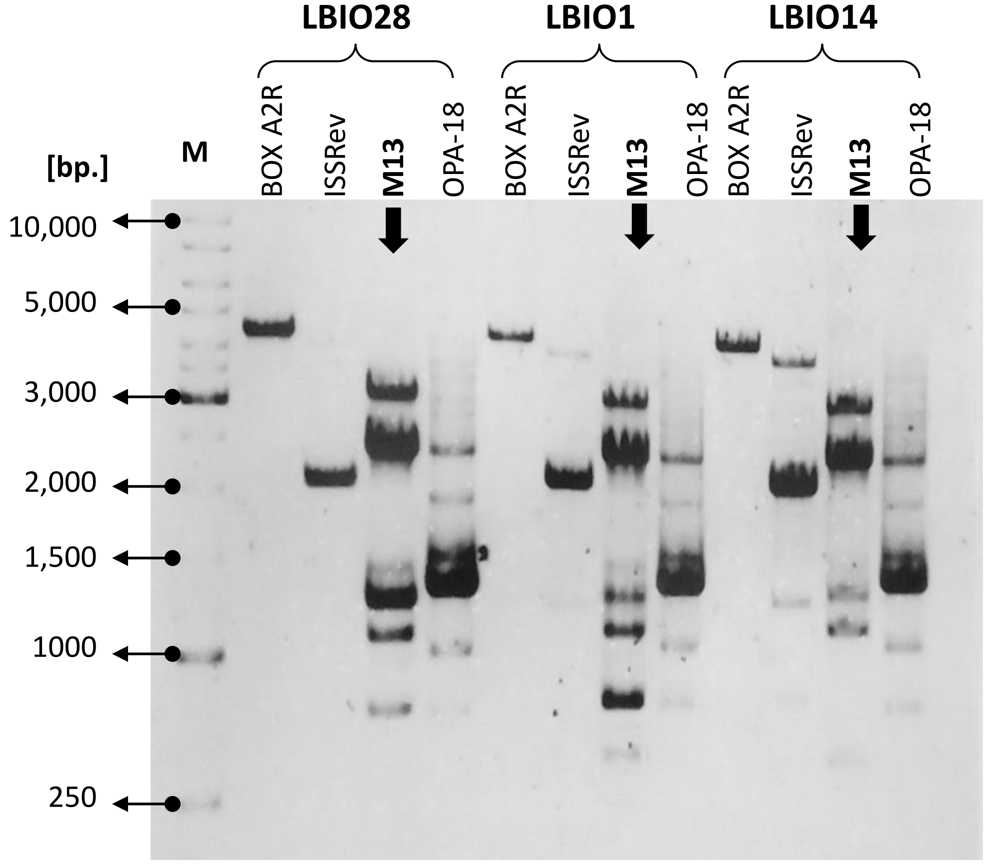

1. Roy, D., Ward, P., Vincent, D. & Mondou, F. (2000) Molecular identification of potentially probiotic lactobacilli. *Cur. Microbiol.* **40**: 40–46. DOI: 10.1007/s002849910008
2. Huey, B., & Hall, J. (1989) Hypervariable DNA fingerprinting in *E. coli* minisatellite probe from bacteriophage M13. *J. Bacteriol.* **171**: 2528–2532.
3. Acedo-Félix, E., & Pérez-Martínez, G. (2003). Significant differences between *Lactobacillus casei* subsp. *casei* ATCC 393 T and a commonly used plasmid-cured derivative revealed by a polyphasic study. *Int. J. Syst. Evol. Microb*. **53**: 67–75. DOI:10.1099/ijs.0.02325-0
4. Nayak, B.S., Badgley, B., & Harwood, V.J. (2011). Comparison of genotypic and phylogenetic relationships of environmental *Enterococcus* isolates by BOX-PCR typing and 16S rRNA gene sequencing. *Appl. Environ. Microbiol*. **77**: 5050–5055. DOI: 10.1128/aem.00130-11

Supplementary Figure S2: SEC-MALLS analysis of the EPS fraction extracted from milks fermented with the *L. plantarum* LBIO1, LIO14 and LBIO28 strains. Detector: ultra violet (UV, 280 nm), multiangle light scattering (LS, angle 90°) and refraction index (RI).

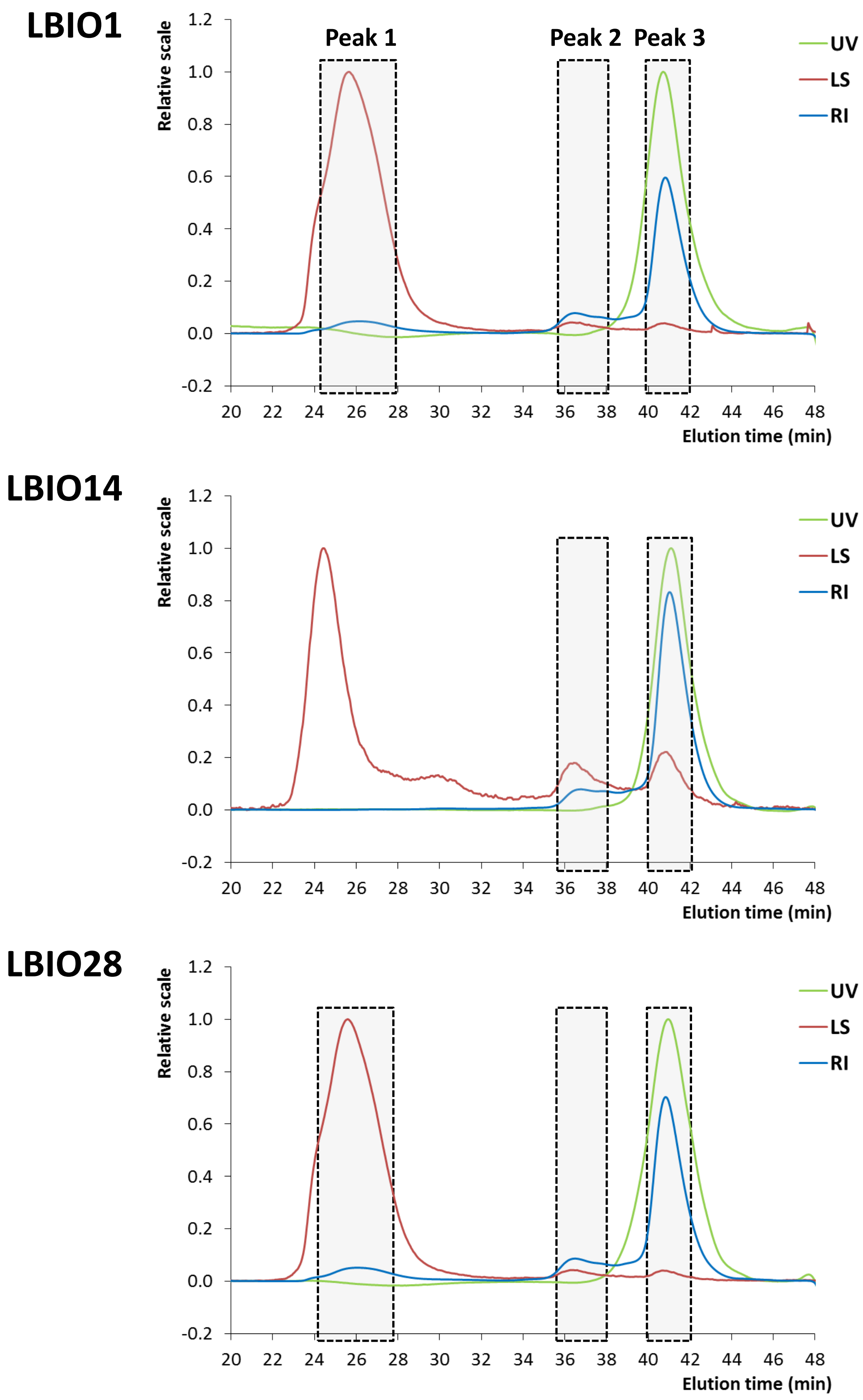

Supplementary Figure S3: GC-MS analysis of the acetylated methyl glycosides, with HF treatment, of hydrolyzed EPS extracted from milks fermented with the *L. plantarum* LBIO1, LIO14 and LBIO28 strains. Monomers: glycerol (Gro), ribose (Rblo), mannose (Man), galactose (Gal), glucose (Glc), galactosamine (GalN), glucosamine (GlcN), and neuraminic acid (NeuA). "*i*" means impurities.

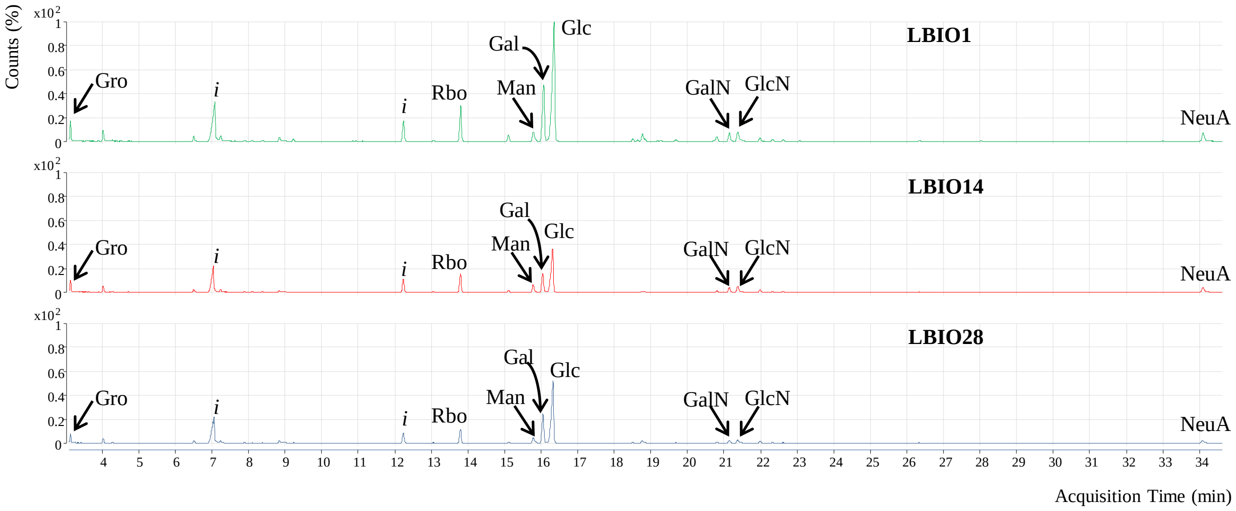

Supplementary Table S1: Rheological parameters of milks fermented with *L. plantarum* LBIO1, LBIO14 and LBIO28 strains following the Ostwald-de Waele flow model (share rate 300 s^{-1}). Differences among strains was determined by ANOVA and the SNK (Student- Newman-Keuls) mean comparison test (p<0.05).

Strain	**Viscosity** (mPa.s^{-1})	**Consistency coefficient (K)** (Pa. s^n)	**Flow behaviour index (n)** (dimensionless)
**LBIO1**	38.17±1.05 ^{b}	3.95±0.24 ^{b}	0.187±0.01 ^{b}
**LBIO14**	19.05±6.17^{a}	1.31±0.46 ^{a}	0.260±0.02 ^{c}
**LBIO28**	50.42±5.83 ^{c}	6.23±1.09 ^{c}	0.156±0-01 ^{a}

Strains that do not share a common superscript are statistically different (p<0.05) according to the SNK test.

Supplementary Table S2: Acidification rate (regression equations of pH vs. time in the 14-24 h range) and increase of bacterial counts (CFU/ml) after 24 h fermentation with respect to the initial counts (0 h) of milks fermented with *L. plantarum* LBIO1, LBIO14 and LBIO28 strains. Differences among strains was determined by ANOVA and the SNK (Student- Newman-Keuls) mean comparison test (p<0.05).

Strain	**Regression equations** (correlation coefficient)	**Slope**	**Bacterial increase** (CFU/ml)
**LBIO1**	y = -0.0955x + 6.9958 (R^2 = 0.9973)	-0.0955±0.0095 $^{\mathbf{a}}$	1.71±0.23 x10^9 $^{\mathbf{b}}$
**LBIO14**	y = -0.0887x + 7.0419; (R^2 = 0.9515)	-0.0887±0.0029 $^{\mathbf{a}}$	1.17±0.21 x10^9 $^{\mathbf{a}}$
**LBIO28**	y = -0.1286x + 7.3508; (R^2 = 0.9990)	-0.1285±0.0078 $^{\mathbf{b}}$	1.41±0.04 x10^9 $^{\mathbf{ab}}$

Strains that do not share a common superscript are statistically different (p<0.05) according to the SNK test.

Comparison with WCFS1 genome (Kleerebezem et al., 2003)

	WCFS1	LBIO1	LBIO28	LBIO14	**locus tag**	**Gene function**
					lp1176	UDP-galactopyranose mutase
cps1 A-I					*lp1177*	polysaccharide biosynthesis protein
					lp1178	glycosyltransferase, family 1 (GT1)
					lp1179	oligosaccharide transporter (flippase)
					lp1180	glycosyltransferase
					lp1181	acyltransferase/acetyltransferase
					lp1182	polysaccharide biosynthesis protein, chain length regulator
					lp1183	glycosyltransferase, family 1 (GT1)
					lp1184	glycosyltransferase (rhamnosyltransferase),family 2 (GT2)
					lp1185	polysaccharide polymerase
					lp1186	rbfA
					lp1187	glycosylhydrolase
					lp1188	rbfC
					lp1189	rbfB
					lp1190	rbfD
					lp1191	transposase fragment
					lp1195	transposase fragment
					lp1196	integrase / recombinase
cps2 A-J				3_0923	*lp1197*	polysaccharide biosynthesis protein, chain length regulator
				3_0924	*lp1198*	polysaccharide biosynthesis protein,regulator
				3_0925	*lp1199*	polysaccharide biosynthesis protein,phosphotyrosine-protein phosphatase
					lp1200	UDP N-acetyl glucosamine 4-epimerase, NAD dependent
				**3_1674**	***lp1201***	priming glycosyltransferase, polyprenyl glycosylphosphotransferase
					lp1202	glycosyltransferase, family 1 (GT1)
					lp1203	polysaccharide biosynthesis protein
					lp1204	polysaccharide polymerase
					lp1205	oligosaccharide transporter (flippase)
					lp1206	glycosyltransferase, family 1 (GT1)

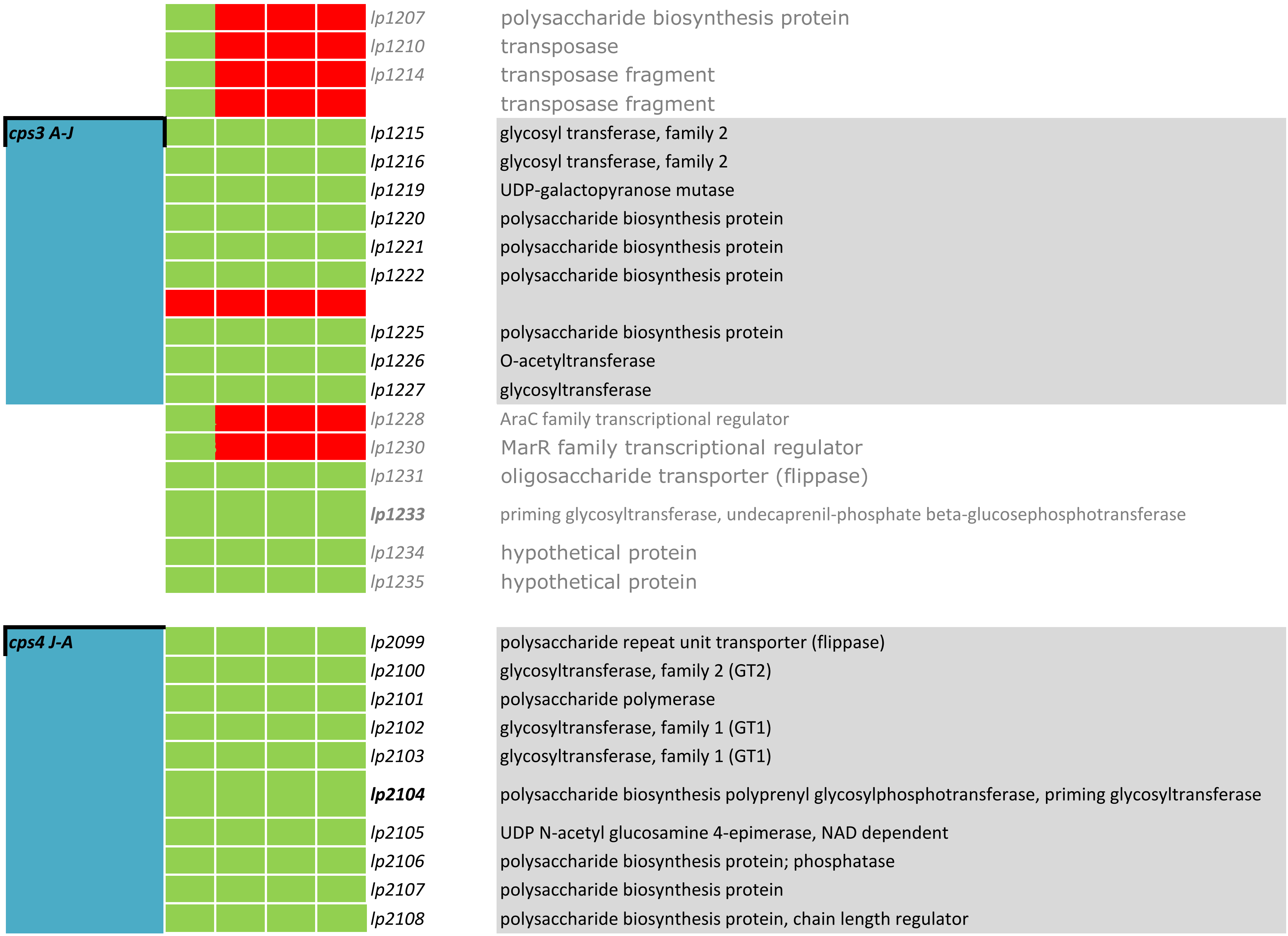

cps3 A-J
lp1207 polysaccharide biosynthesis protein
lp1210 transposase
lp1214 transposase fragment
transposase fragment
lp1215 glycosyl transferase, family 2
lp1216 glycosyl transferase, family 2
lp1219 UDP-galactopyranose mutase
lp1220 polysaccharide biosynthesis protein
lp1221 polysaccharide biosynthesis protein
lp1222 polysaccharide biosynthesis protein
lp1225 polysaccharide biosynthesis protein
lp1226 O-acetyltransferase
lp1227 glycosyltransferase
lp1228 AraC family transcriptional regulator
lp1230 MarR family transcriptional regulator
lp1231 oligosaccharide transporter (flippase)
lp1233 priming glycosyltransferase, undecaprenil-phosphate beta-glucosephosphotransferase
lp1234 hypothetical protein
lp1235 hypothetical protein
cps4 J-A
lp2099 polysaccharide repeat unit transporter (flippase)
lp2100 glycosyltransferase, family 2 (GT2)
lp2101 polysaccharide polymerase
lp2102 glycosyltransferase, family 1 (GT1)
lp2103 glycosyltransferase, family 1 (GT1)
lp2104 polysaccharide biosynthesis polyprenyl glycosylphosphotransferase, priming glycosyltransferase
lp2105 UDP N-acetyl glucosamine 4-epimerase, NAD dependent
lp2106 polysaccharide biosynthesis protein; phosphatase
lp2107 polysaccharide biosynthesis protein
lp2108 polysaccharide biosynthesis protein, chain length regulator

Capsular polysaccharide clusters (Remus *et al.* 2012)

WCFS1	ORF		Gene name(s)	Gene function
	lp_1176	*Glf*		UDP-galactopyranose mutase
cps1 A-I	*lp_1177*	*cps1A*		Glycosyltransferase
	lp_1178	*cps1B*		glycosyltransferase, family 1 (GT1)
	lp_1179	*cps1C*	*wzx*	oligosaccharide transporter (flippase)
	lp_1180	*cps1D*		Glycosyltransferase
	lp_1181	*cps1E*		acyltransferase/acetyltransferase
	lp_1182	*cps1F*	*wzz/wzd*	polysaccharide biosynthesis protein, chain length regulator
	lp_1183	*cps1G*		glycosyltransferase, family 1 (GT1)
	lp_1184	*cps1H*		glycosyltransferase, family 2 (GT2)
	lp_1185	*cps1I*	*wzy*	polysaccharide polymerase
rfb A-D	*lp_1186*	*rfbA*		glucose-1-phosphate thymidylyltransferase
	lp_1187			glycosyl hydrolase
	lp_1188	*rfbC*		dTDP-4-dehydrorhamnose 3,5-epimerase
	lp_1189	*rfbB*		dTDP-glucose 4,6-dehydratase
	lp_1190	*rfbD*		dTDP-4-dehydrorhamnose reductase
	lp_1191			transposase, fragment
	lp_1195			transposase, fragment
	lp_1196			integrase/recombinase
cps2 A-J	*lp_1197*	*cps2A*	*wzd*	polysaccharide chain-length regulator
	lp_1198	*cps2B*	*wze*	polysaccharide chain-length regulator; tyrosine kinase
	lp_1199	*cps2C*	*wzh*	polysaccharide chain-length regulator; tyrosine phosphatase
	lp_1200	*cps2D*		UDP N-acetylglucosamine 4-epimerase, NAD dependent
	lp_1201	*cps2E*		priming glycosyltransferase, polyprenyl glycosylphosphotransferase
	lp_1202	*cps2F*		glycosyltransferase, family 1 (GT1)
	lp_1203	*cps2G*		Glycosyltransferase
	lp_1204	*cps2H*	*wzy*	polysaccharide polymerase
	lp_1205	*cps2I*	*wzx*	oligosaccharide transporter (flippase)
	lp_1206	*cps2J*		glycosyltransferase, family 1 (GT1)

	lp_1207	*cps2K*		polysaccharide biosynthesis protein
	lp_1210			hypothetical protein
	lp_1214			transposase, fragment
				transposase, fragment
cps3 A-J	*lp_1215*	*cps3A*		glycosyltransferase, family 2 (GT2)
	lp_1216	*cps3B*		glycosyltransferase, family 2 (GT2)
	lp_1219	*glf*		UDP-galactopyranose mutase
	lp_1220	*cps3D*		polysaccharide biosynthesis protein
	lp_1221	*cps3E*		polysaccharide biosynthesis protein; putative protein kinase
	lp_1222	*cps3F*	*wzy*	polysaccharide biosynthesis membrane protein
	lp_1224	*cps3G*	*wzy*	polysaccharide polymerase, partial
	lp_1225	*cps3H*		polysaccharide biosynthesis protein
	lp_1226	*cps3I*		O-acetyltransferase
	lp_1227	*cps3J*		Glycosyltransferase
	lp_1228			transcription regulator, AraC family
	lp_1230			transcription regulator, MarR family
	lp_1231			oligosaccharide transporter (flippase)
	lp_1233			priming glycosyltransferase, undecaprenyl-phosphate beta-glucosephosphotransferase
	lp_1234			hypothetical protein
	lp_1235			hypothetical protein

cps4 J-A	*lp_2099*	*cps* 4J	wzx	polysaccharide repeat unit transporter (flippase)
	lp_2100	*cps* 4I		glycosyltransferase, family 2 (GT2)
	lp_2101	*cps* 4H	wzy	polysaccharide polymerase
	lp_2102	*cps* 4G		glycosyltransferase, family 1 (GT1)
	lp_2103	*cps* 4F		glycosyltransferase, family 1 (GT1)
	lp_2104	*cps* 4E		polysaccharide biosynthesis polyprenyl glycosylphosphotransferase, priming glycosyltransferase
	lp_2105	*cps4D*		UDP N-acetylglucosamine 4-epimerase, NAD dependent
	lp_2106	*cps* 4C	wzh	polysaccharide chain-length regulator; tyrosine phosphatase
	lp_2107	*cps* 4B	wze	polysaccharide chain-length regulator; tyrosine kinase
	lp_2108	*cps* 4A	wzd	polysaccharide chain-length regulator

Comparison with WCFS1 genome

WCFS1 locus tag	LBIO1 ORF	LBIO1 % Identity	LBIO28 ORF	LBIO28 % Identity	LBIO14 ORF	LBIO14 % Identity
lp1176						
lp1177	*HJQ46_14785*	86.36	*HIG33_14745*	86.38	*HIF97_14930*	86.38
lp1178						
lp1179						
lp1180						
lp1181						
lp1182						
lp1183						
lp1184						
lp1185						
lp1186						
lp1187						
lp1188						
lp1189						
lp1190						
lp1191						
lp1195						
lp1196						
lp1197	*HJQ46_11970*	92.67	*HIG33_14845*	92.67		
lp1198	*HJQ46_11965*	96.28	*HIG33_14840*	96.28		
lp1199	*HJQ46_11960*	91.44	*HIG33_14835*	91.44		
lp1200						
lp1201	*HJQ46_11955*	40.69	*HIG33_14830*	40.69		
lp1202						
lp1203						
lp1204						
lp1205						
lp1206						

lp1207						
lp1210						
lp1214						
lp1215	HJQ46_13005	70.20	HIG33_12980	70.20	HIF97_12335	70.20
lp1216	HJQ46_13000	98.71	HIG33_12975	98.71	HIF97_12330	98.71
lp1219	HJQ46_12995	99.20	HIG33_12970	99.20	HIF97_12325	99.20
lp1220	HJQ46_12990	99.14	HIG33_12965	99.14	HIF97_12320	99.14
lp1221	HJQ46_12985	98.07	HIG33_12960	98.07	HIF97_12315	98.07
lp1222	HJQ46_12980	98.65	HIG33_12955	98.65	HIF97_12310	98.65
lp1224	HJQ46_12980	97.00	HIG33_12955	97.00	HIF97_12310	97.00
lp1225	HJQ46_12975	97.41	HIG33_12950	97.41	HIF97_12305	97.41
lp1226	HJQ46_12970	97.77	HIG33_12945	97.77	HIF97_12300	97.77
lp1227	HJQ46_12965	99.61	HIG33_12940	99.61	HIF97_12295	99.61
lp1228						
lp1230						
lp1231	HJQ46_12960	93.01	HIG33_12935	93.01	HIF97_12290	93.01
lp1233	HJQ46_12955	99.55	HIG33_12930	99.55	HIF97_12285	99.55
lp1234	HJQ46_12950	99.01	HIG33_12925	99.01	HIF97_12280	99.01
lp1235	HJQ46_12945	100.00	HIG33_12920	100.00	HIF97_12275	100.00
lp2099	HJQ46_12370	99.17	HIG33_12345	99.17	HIF97_09330	99.17
lp2100	HJQ46_12375	98.14	HIG33_12350	98.14	HIF97_09335	98.14
lp2101	HJQ46_12380	97.17	HIG33_12355	97.17	HIF97_09340	97.17
lp2102	HJQ46_12385	94.40	HIG33_12355	94.44	HIF97_09345	94.44
lp2103	HJQ46_12390	94.55	HIG33_12365	94.55	HIF97_09350	94.55
lp2104	HJQ46_12395	99.55	HIG33_12370	99.55	HIF97_09355	99.55
lp2105	HJQ46_12400	100.00	HIG33_12375	100.00	HIF97_09360	100.00
lp2106	HJQ46_12405	99.62	HIG33_12380	99.62	HIF97_09365	99.62
lp2107	HJQ46_12410	98.72	HIG33_12385	100.00	HIF97_09370	98.72
lp2108	HJQ46_12415	98.41	HIG33_12390	98.41	HIF97_09375	98.41
